# Keratin from Animal By-Products: Structure, Characterization, Extraction and Application—A Review

**DOI:** 10.3390/polym16141999

**Published:** 2024-07-12

**Authors:** Shahin Banasaz, Vincenza Ferraro

**Affiliations:** INRAE, QuaPA, 63122 Saint-Genès-Champanelle, France; shahin.banasaz@inrae.fr

**Keywords:** keratin, animal by-products, characterization, extraction, valorization

## Abstract

Keratin is a structural fibrous protein and the core constituent of animal by-products from livestock such as wool, feathers, hooves, horns, and pig bristles. This natural polymer is also the main component of human hair and is present at an important percentage in human and animal skin. Significant amounts of keratin-rich animal tissues are discarded worldwide each year, ca. 12 M tons, and the share used for keratin extraction and added-value applications is still very low. An important stream of new potential raw materials, represented by animal by-products and human hair, is thus being lost, while a large-scale valorization could contribute to a circular bioeconomy and to the reduction in the environmental fingerprint of those tissues. Fortunately, scientific research has made much important progress in the last 10–15 years in the better understanding of the complex keratin architecture and its variability among different animal tissues, in the development of tailored extraction processes, and in the screening of new potential applications. Hence, this review aims at a discussion of the recent findings in the characterization of keratin and keratin-rich animal by-product structures, as well as in keratin recovery by conventional and emerging techniques and advances in valorization in several fields.

## 1. Introduction

Animal livestock by-products are rich in organic compounds, and some streams are significantly rich in structural fibrous proteins, also called scleroproteins, from the Greek word for “hard, insoluble proteins” [1,2,3,4]. These proteins are natural polymers that have mechanical and protective roles and no purpose in nutrition [5,6]. Among all animal by-products (as defined by the European regulation EC No. 1069/2009), non-edible tissues, such as feathers, wool, hooves, horns, claws, beaks, pig bristles, bovine hide hairs, and fish scales, are essentially constituted by keratin [1,2]. In humans and animals, this scleroprotein has a variety of functions such as waterproofing, regulation of temperature, cohesion and structuring of tissues, cushioning to protect the deeper tissues against mechanical shocks and infection, wound healing, nerve repair, and excretion of wastes and toxins from the integumentary system (skin and its appendages) [5,7]. In terms of its biological importance, keratin is the most abundant structural protein in epithelial cells, and it is the most abundant protein after collagen, both in humans and animals [2,5,6]. The remarkable mechanical and biological properties of keratin can be valorized in several applications, such as bio-based materials (e.g., bioplastics, anti-fire and antimicrobial coatings, edible casings), biomaterials (e.g., tissue and nerve repair and regeneration), pharmaceuticals (e.g., drug coatings for controlled release, anti-inflammatory products), cosmetics (e.g., hair and skin care), functional foods (e.g., fiber analogs), textiles (e.g., functionalized wearables), and agricultural products (e.g., biostimulants, coatings for fertilizers) [1,3,4,8,9]. Furthermore, in terms of its potential, keratin can contribute to satisfying the significant demand for biopolymers and bioplastics, forecasted to increase by 24.2% over the period 2024–2029 [10]. The analysis of the scientific literature has revealed that, in the period 2001–2020, the number of publications on keratin from natural sources has increased more than 10-fold with respect to the preceding period of 1980–2020 (INRAE QuaPA unit analysis of 2022 on the databases Scopus, Web of Science, PubMed, Google Scholar, World International Patent Office, Google Patents), which underlies the sharp expansion of the interest in this natural polymer. Nevertheless, the exploitation of keratin’s potential is lagging because of several factors: few high-value applications have been implemented outside the laboratory (mainly keratin peptides in cosmetics); most of the recovery processes are not fully tailored to the complexity of the keratin structure so as to harness its functionalities; and, until now, animal-derived natural polymers have been overlooked with respect to plant ones, in spite of their unique properties. At the same time, significant amounts of keratin-rich animal by-products are generated each year: in 2020, the annual world production was ca. 12 million tonnes [1]. Major sources of keratin are chicken, duck, goose, and turkey feathers (90% constituted of keratin by weight), sheep and goat wool (95% keratin), and pig bristles (95% keratin) [1,3,4]. However, this potential stream of “raw materials” is essentially wasted. In the European Union, keratinous by-products must be treated in specialized sites by incineration or landfill; some by-products, such as wool, can be burned or buried at the farm only if previously disinfected (EC Regulation 1069/2009). However, these treatments, apart from their often unauthorized handling, lead to the production and release of significant amounts of harmful gases such as CO, CO_2_, SO_x_, and NO_x_ [11,12].

In this context, this review aims to present, in a comprehensive manner, keratin-rich animal by-products and their valorization for high-value applications with increased economic impact and reduced environmental fingerprint with respect to the current management. Main and crucial aspects are discussed, such as the complexity of keratin and animal keratinous tissues’ structure, their characterization by currently available techniques, the progress in keratin extraction methodologies, and advances in their application.

## 2. Keratin Structure

Currently, the term “keratins” identifies a group of insoluble and heterogeneous filament-forming proteins produced by specific epithelial cells of vertebrates [5]. Keratins are synthesized and regulated by the messenger ribonucleic acid (RNA) inside keratinocytes; after cell division and maturity, the production of RNA and proteins stops, the degradation of the nucleus starts, and, finally, the cell dies, filled with keratin [5,13]. Hence, post-synthetic chemical modifications such as the formation of disulfide linkages between several keratin molecules occur through two cysteine residues. This produces a stabilization of the protein network, which can be organized into two main configurations, namely α-keratin and β-keratin, and can exhibit different cross-linking degrees and different mechanical behaviors [5,6,13]. Originally, the term “keratin” was used around the year 1850 to identify the hard and insoluble material constituting animal horns, from the Greek “*kέraς*”, although the first use of this protein was reported to be in the 16th century by a Chinese herbalist who developed medical applications from pyrolyzed human hair [14]. In the 20th century, there was increasing interest in keratin extracted from animal and human hair for biological applications such as powders for cosmetics and coatings for drugs; several other tissues, such as animal skin, wool, feathers, and hooves, were also used [6,8,9]. However, with the technical advances of the 1920s, it was observed that the keratin product recovered was actually a mixture of keratins, represented by several variants of keratin, keratin filament-forming proteins, and other proteins such as enzymes [4,7,9,13]. An initial nomenclature was also proposed by naming the filament-forming proteins that were extracted from the cornified (dead) layer of the epidermis “keratin” and naming the proteins extracted from the living layers of the epidermis “prekeratins” or “cytokeratins” [8,9,13]. The advances in extraction methods and characterization techniques in the 1970s allowed for significant new knowledge on the amino acid sequence and supramolecular structure of several keratin variants, as well as on the assembly of keratin giving rise to keratinous tissues. In 1982, a comprehensive nomenclature was established based on the keratin proteins that were known at that time, mainly of human origin; that was finally updated in 2006, thanks to the advances in genomic studies; 54 keratin proteins have been identified in mammals, and they are very similar in humans, cattle, and sheep [5,6]. 

Whatever the keratin variant and the keratinous tissue, either in humans or animals, from a structural point of view, keratins are consensually and more than often classified as α-keratin and β-keratin, based on X-ray diffraction [4,6,7,15]. Both are complex hierarchical structures with dimensions spanning from the nanometer to the centimeter; they can further be arranged into tubular or sandwich structures, giving rise to several skin appendages [6,7,8,9]. The presence of disulfide bonds at several levels of the keratin architecture is the basis of its mechanical properties and stability towards many enzymes [6,7]. In both α-keratin and β-keratin, the number and sequence of amino acids, the polarity, the charge, and the chain length can vary; their properties are extremely dependent on the sequence of amino acids, where even a small modification can have a significant effect [4,8]. In the following sessions, α-keratin and β-keratin are discussed in detail; their main and distinctive features are reported in Table 1.

### 2.1. α-Keratin

**Figure 1:**
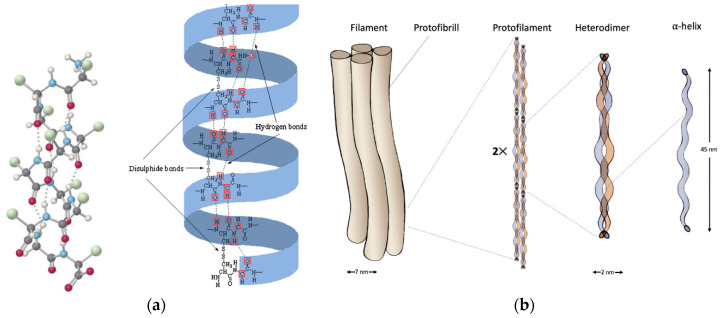
Intermediate filament structure of α-keratin: (**a**) the α-helical structure of wool keratin with the location of hydrogen bonds (red boxes connected with a dotted line) (**b**) the formation of intermediate filament (image from [3,6] with permission).

α-keratin is found in mammals and is the primary component of hair, nails, wool, hooves, horns, and stratum corneum. At the smallest scale, the nanometer, it shows a fine filament structure called intermediate filament (IF) of 7–10 nm in diameter [6,14,15,16]. The term “intermediate” is relative to the fact that α-keratin diameter is between two other major classes of filamentous structures, namely microfilaments (such as actin, 7 nm) and microtubules (24 nm) [7,13]. The IFs are not polarized; hence they do not participate in cellular functions but act as a scaffold for the cytoskeleton [7]. They have an ordered and crystalline structure, organized as coiled coils based on the α-helix configuration, common to other structural proteins such as collagen and elastin [2,6]. Two right-handed α-helix chains are wound by disulfide crosslinks from two molecules of cysteine, to form a left-handed coiled coil, which represents the dimer, the molecular unit of α-keratin, 45 nm long [2,14,16]. The dimers are linked end-to-head by a peptide bond between the non-helical nitrogen and carbon domains and side-to-side by disulfide bridges to form a protofilament of ca. 2 nm in diameter. Two protofilaments associate laterally to form a protofibril, and finally, four protofibrils associate with each other to form a tightly packed helical IF with a diameter of 7 nm (Figure 1). Once the IF is formed, it links with the matrix proteins through the non-helical C- and N-terminal domains. The matrix is amorphous and consists of a fraction rich in sulfur from cysteine, and another fraction rich in glycine, tyrosine, and phenylalanine; the ratio matrix/IF varies according to the tissue, e.g., 0.42 in wool and 0.54 in human hair [6,17,18]. The α-keratin protofilament is composed of ca. 380–620 amino acids, corresponding to a molecular mass between 40 and 70 kDa [5,7,18]. The amino acid residues can vary from one animal species to another (Table 2); nonetheless, whatever the species, the central helical domain of α-keratin is always composed of hydrophobic residues (alanine, glycine, leucine, proline, and valine), while acid and basic amino acids are distributed at the N- and C-terminal domains [7,18]. The alignment of the IF can also vary according to the species, and it influences the mechanical properties, where the higher the order of alignment, the higher the tensile strength [6,13,18].

### 2.2. β-Keratin

**Figure 2:**
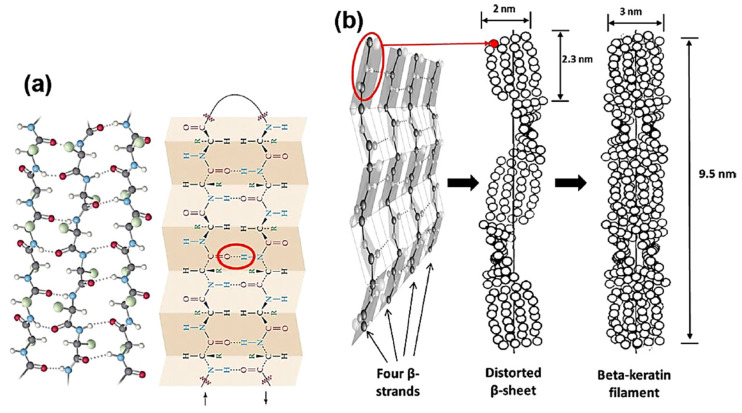
(**a**) Pleated β-sheet and (**b**) assemblage of two distorted β-sheets, of four β-strands each, to form a β-keratin filament; ellipse in red represents hydrogen bonds (image from [6] with permission).

β-keratin is mainly found in avians, where it is the main constituent of feathers, beaks, and claws; non-livestock by-products such as reptilian scales, corneous materials of turtle carapaces, and turtle plastron are also made of β-keratin [6,19]. At the nanometric scale (the smallest level), β-keratin also has a filamentous structure, which is called beta-keratin filament, with a diameter of 3–4 nm. The filament is based on a β-sheet conformation: four β-strands assemble laterally either in parallel or antiparallel (which is more stable) to form a β-sheet; the strands are held together by hydrogen bonds that force the sheet to distort on a left-handed helical surface [20,21], while the peptide bonds in the polypeptide chain force the sheet to pleat [6,19]. Two β-sheets assemble to form a β-keratin filament (the molecular unit of β-keratin) (Figure 2), which is ca. 9.5 nm long and has a molecular mass of 10–22 kDa [21,22]. Two C- and N-terminal polypeptides are linked to the central β-sheet structure; they are amorphous and represent the matrix that winds around the filament domain to form the keratin network [20,21,22].

The amino acid composition varies among species and is different from α-keratin (examples in Table 2 and Table 3). As for the α-keratin, in the β-keratin, the hydrophobic domain is also located in the central pleated zone, while the terminal N- and C-domains are constituted by acidic and basic residues [6,20,21]. From a mechanical point of view, β-keratin is less extensive than α-keratin [16,20]; it has also been observed that under a tensile load between 2–5% and 30–50%, an irreversible transition of keratin from α-helix to β-sheet occurs [6].

**Table 1:** Features of α-keratin and β-keratin [6,7,18,19,20,21,22].

Feature	α-Keratin	β-Keratin
Common structure	Filament matrix: the filaments are embedded into an amorphous matrix	
Occurrence	Wool, hair, stratum corneum, fingernails, horns, hooves, quills	Feathers, beaks, and claws; reptilian scales; turtle carapaces and plastron
Type of filaments and diameter	Intermediate filaments (IFs), 7 nm	Beta-keratin filaments, 3–4 nm
Constituting proteins	The IFs can be several kinds of low-sulfur proteins while the matrix is made of high-sulfur and high-glycine–tyrosine proteins	The filament and the matrix are incorporated into one single protein
Synthesis	In the beginning, IFs (low-sulfur) are synthesized; as the cell approaches maturation, matrix proteins (high-sulfur) are produced between the IFs and after the synthesis takes place concomitantly	There are no different synthesis stages; filaments and matrix increase in a coordinated way; the mechanism of aggregation is not known in detail
Molecular unit (MU)	Dimer	Distorted pleated sheet
Protofilament molecular mass	40–68 kDa	10–22 kDa
Number of residues in the MU	33–35 for the helical zone, 136 for the non-helical zone	34 for the pleated sheet, 59–168 for the non-sheet zone
Mechanical properties	α-keratin has lower stiffness than β-keratin	
	α-helix changes into β-pleated sheet under tension	
	Young’s modulus for α- and β-keratin decreases with an increase in humidity	
	Mineralization with calcium can harden both keratins	
	Two-phase model: crystalline and water-resistant IFs in an amorphous matrix that is modeled as an elastomer that can interact with water	Crystalline filaments wound by an amorphous matrix. (only a few studies are available)

**Table 2:** Amino acid composition of keratin in several animal by-products (mol%).

Amino Acid (mol%)	Feathers (Whole) (β-Keratin)	Feather (Rachis) (β-Keratin)	Wool (Sheep) (α-Keratin)	Horn (Sheep) (α-Keratin)	Hoof (Sheep) (α-Keratin)	Bristles (Pig) (α-Keratin)
Alanine	3.60	8.7	5.20	5.90	6.37	4.90
Arginine	5.40	3.8	6.24	6.68	7.16	7.65
Aspartic acid	4.70	5.6	5.93	7.80	8.39	6.05
half-Cystine	7.70	7.8	13.10	6.24	5.66	10.75 *
Glutamic acid	7.70	6.9	11.10	12.90	13.70	12.55
Glycine	6.20	13.7	8.56	11.10	9.10	9.25
Histidine	nr	0.2	0.79	1.33	0.94	nr
Isoleucine	4.30	3.2	2.98	3.31	3.56	3.15
Leucine	7.00	8.3	7.20	9.13	9.51	6.95
Lysine	0.60	0.6	2.66	3.76	3.96	2.60
Methionine	1.30	0.1	0.54	0.81	0.80	0.65
Phenylalanine	4.20	3.1	2.48	2.64	2.65	2.30
Proline	8.70	9.8	6.60	3.83	3.99	7.15
Serine	9.30	14.1	10.80	9.56	9.54	11.30
Threonine	3.50	4.1	6.53	4.78	4.95	6.95
Tyrosine	1.95	1.4	3.78	5.00	4.03	3.85
Valine	6.94	7.8	5.68	5.21	5.66	4.85
	[2]	[6]	[23]	[23]	[23]	[24]

* S-Carboxymethylcysteine; nr: not reported.

### 2.3. Other Keratin Classifications 

Keratins are also classified based on the isoelectric point (pI), the content of sulfur, and the species [13,18,25]. According to their pI (i.e., the pH at which the proteins are neutral), keratins are classified as acidic, also called type I or subfamily A (pI of 4.9–5.6), and basic, or type II or subfamily B (pI of 6–8.5). This classification has been proposed mainly for mammals, for which 54 keratin variants have been identified: 28 of type I (17 epithelial and 11 hair/wool keratins) and 26 of type II ( 20 epithelial and 6 hair/wool) [6]. 

Based on the content of sulfur, ‘soft’ and ‘hard’ keratins can occur, and these can be either α- or β-keratins. In soft keratin, the filament bundle has a cysteine content of less than 3% (of the total amino acid residues), and the filaments themselves are loosely packed; tissues with soft keratin, namely the skin stratum corneum, are flexible and act as a barrier to external agents and also camouflage from predators [7,9,16]. Hard keratins consist of filaments that are embedded in a cysteine-rich amino acid matrix (ca. 6–16% of the total amino acid residues), resulting in a high degree of sulfur crosslinking with respect to soft keratins; hard keratins also have higher amounts of lipids. Tissues made of hard keratin, such as hair and wool, pig bristles, horns, nails and hoofs, beaks, claws, and feather shafts, are more resistant to heat, enzymes, and both oxidizing and reducing agents than soft keratin. In addition, hard α-keratin is 400–800 times stiffer than soft α-keratin [7,9,16]. Fraser and Macrae [13] proposed the class of γ-keratins (apart from the α- and β-keratins), identified as non-structural and acid-soluble keratins. Some later studies showed that this is a class of α-keratin-derived proteins instead, obtained from an oxidative extraction of primary fragments of α-keratin itself [5,6]. Other authors identified γ-keratins as the matrix proteins of the keratin network [2,6]. Finally, keratin is sometimes discussed as mammal, avian, and reptilian keratin, based on the specific biology and evolution of each animal group [6,13,16]. 

### 2.4. Structure of Keratinous Livestock By-Products

Keratinous tissues are formed through the production, differentiation, and maturation of keratinocytes [6,13]. After cell production, the process of keratinization replaces the cytoplasm with filamentous proteins, giving rise to dead cells with a stable structure. Later, several ultrastructural changes of keratin occur, together with the formation of new cells with different shapes and growing directions. In turn, these cells will undergo the maturation process (keratinization); interaction with non-keratinous materials (such as minerals, lipids, and enzymes) will occur, and, layer by layer on an increasing length, a new tissue is formed [5,6,13]. According to specific functions, several keratinous tissues are produced: hooves and feathers to guarantee motion; horns, beaks, and claws for defense; wool, hair, and bristles for skin protection and temperature regulation [5,25]. In the following paragraphs, the main keratinous animal by-products are discussed. 

#### 2.4.1. Wool Keratin

**Figure 3:**
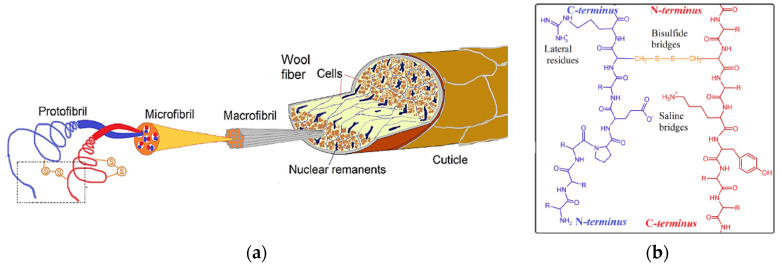
Wool fiber structure (**a**) and chemical interactions between keratin chains (protofibrils) giving rise to the fiber (**b**) (image from [29] with permission).

Among all animal by-products, wool is by far the most prominent in virtue of its main usage in textile applications for a long time. Australia is the first producer of wool from livestock sheep and goats; 420,000 tonnes/year are generated, which represents about one-quarter of the world quantity [23,26]. On a weight basis, wool is constituted by ca. 95% keratin, ca. 1% waxy lipids (incorporating vitamin D3), a small number of polysaccharides, and water [23,27]. Wool is a hard-keratinous material tightly packed through a high degree of disulfide crosslinking, hydrogen bonds, and salt bonds (Figure 3); α-helical filaments are embedded in an amorphous β-sheet protein matrix to give rise to a wool fiber diameter of ca. 20 μm [6,27]. The filament and the matrix constitute two main protein fractions, based on the content of sulfur (from carboxymethylcysteine (cys(cm)): high-sulfur proteins, which have a cys(cm) content of ca. 22% and constitute the matrix, and low-sulfur proteins, which have a cys(cm) content of around 6% and constitute the IFs [23,28]. The low-sulfur-containing fraction represents ca. 17% of the total protein [6]. The high-sulfur fraction is also characterized by higher amounts of proline, serine, and threonine and a smaller amount of lysine and histidine with respect to the low-sulfur fraction [23,28]. The amino acid composition of wool keratin is reported in Table 2. From a mechanical point of view, wool has a specific strength (tensile strength/density) of 150 to 260 MPA (at 0 and 100% relative humidity, respectively), which compares to that of stainless steel [6].

#### 2.4.2. Feather Keratin

Feathers are the most important keratinous by-product in terms of quantities, which, moreover, are increasing because of a growing consumption of poultry products [30,31]. It is estimated that 4.7 million tonnes are generated worldwide, each year, from broiler poultry (meat production), and to that should be added the layer of poultry feathers [30]. 

**Figure 4:**
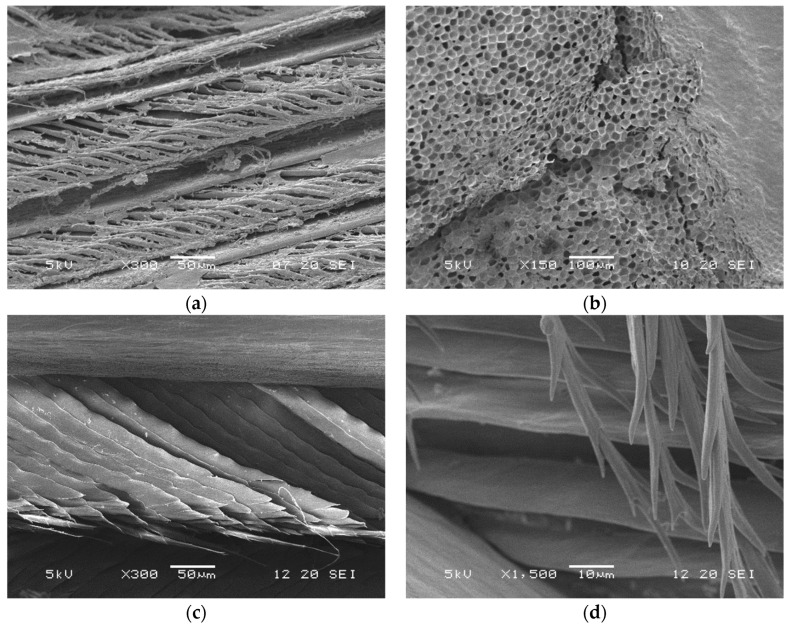
Scanning electron microscopy (SEM) micrographs (INRAE, QuaPA) of the feather shaft medulla (**a**), of the shaft surface (**b**), of the barb (**c**), and of the barbules (**d**) for white chicken.

Feathers are one of the most complex structures in vertebrates, and feather keratin is very similar to the reptilian one [32,33]. Feathers are composed of ca. 90% keratin, ca. 3% fat, ca. 1% ash, and water [6,30]; X-ray studies showed that the dominant keratin secondary structure is the β-sheet [6,32]. From a morphological point of view, three different zones are present: (i) the shaft, (ii) the barb, and (iii) the barbules. The shaft represents the supporting structure of the feather. It is stiff, and it has the structure of a hollow tube (the cortex) (Figure 4a) that contains a foamy material (the medulla) (Figure 4b); it has a medium length of ca. 12–16 cm, and a diameter of 0.3–0.5 cm at the base and ca. 0.1 cm at the top. The shaft can be divided into two zones: the calamus, which anchors the feather to the skin, and the rachis, which supports barbs (Figure 4c) and barbules (Figure 4d). The barbs represent the second-level structure of the feather; they are attached to the rachis and have a length spanning from 1 to 4.5 cm and a diameter of ca. 50 μm. The barbules represent the third-level structure, which develops starting from the barb; they have a length of 0.3–0.5 mm and a diameter of ca. 20 μm [6,32,33]. On a weight basis, about 50% of a feather is the barb, and the other 50% is the shaft [30]. The amino acid content of a whole feather can vary with the breed, the age, the food, and the environment of the poultry [34]. Some data for broiler chickens are reported in Table 3, where the terms ‘white’ and ‘black’ refer to several broiler breeds characterized by white and black feathers, respectively. The amino acid composition of the rachis and barbs is not the same. A higher amount of alanine, glycine, proline, serine, valine, and leucine are found in the rachis (Table 2); significantly higher values for glycine and serine can be noticed, and this is probably explained by the fact that serine is a precursor of cysteine for the rachis and also for the barbs and barbules that develop from the rachis itself. Macro- and micronutrients such as phosphorous, calcium, magnesium, iron, zinc, and copper can also be found in whole feathers [35]. From a mechanical point of view, feathers have the lowest density (0.8 g/cm^3^) among all natural fibers, such as wool (1.3 g/cm^3^) and cellulose (1.5 g/cm^3^) [33].

**Table 3:** Amino acid composition (mol%) of broiler chicken feathers.

Amino Acid (mol%)	White	White	Black	White Crossed Strains
Alanine	4.01	3.90	4.33	2.90
Arginine	6.16	6.58	5.10	6.80
Aspartic acid	5.23	6.15	5.20	4.20
half-Cystine	7.16	7.60	10.54	6.60
Glutamic acid	8.76	10.34	7.75	8.20
Glycine	6.31	6.87	6.80	5.20
Histidine	0.40	0.52	0.32	0.20
Isoleucine	4.28	4.78	3.22	3.90
Leucine	7.38	7.75	6.86	5.70
Lysine	1.11	1.69	0.80	1.60
Methionine	0.25	0.57	0.19	0.70
Phenylalanine	4.40	4.52	3.93	3.50
Proline	8.84	9.37	7.67	nr
Serine	8.93	11.44	8.91	nr
Threonine	3.77	4.66	3.51	3.50
Tryptophan	0.97	2.17	0.94	nr
Tyrosine	2.44	nr	2.12	nr
Valine	6.12	6.30	6.19	5.30
	[36]	[37]	[36]	[30]

nr: not reported.

#### 2.4.3. Hoof and Horn Keratin

**Figure 5:**
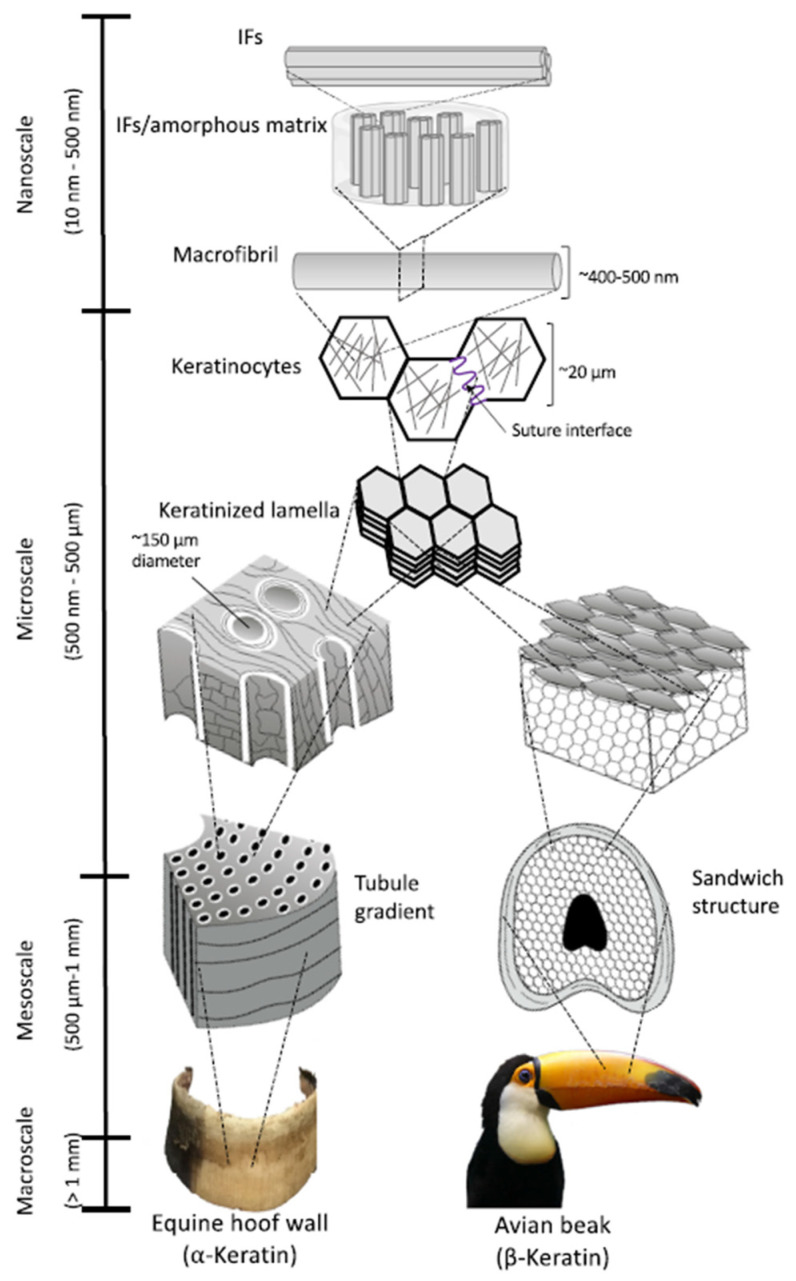
Assemblage of the lamellar and tubular structure of equine hoof (on the left) and comparison with that of avian beak (on the right) (image from [38] with permission).

Hooves and horns belong to the group of horny (hard) keratins, together with nails, claws, and quills [23,38]. The European livestock group of cattle, sheep, and goats generates ca. 87,000 tonnes of horns and ca. 85,000 tonnes of hooves each year (estimated from the livestock population [39]). Both hooves and horns have a composite structure; they are generated by flattened and curved epithelial cells filled with keratin. Two orthogonal assemblages are found in the hoof: circular lamellae, constituted by the cells, concentrically surround longitudinal tubules of the diameter of 200–300 μm, while a macroscale amorphous matrix fills the space between tubules and stacks orthogonally to them (Figure 5, left) [38,40]. Horn has a tri-dimensional layered structure with a porosity gradient along the transverse axis due to the presence of tubules of 40–100 μm diameter [6,38]. The lamellae are aligned longitudinally, and in parallel to the tubules; they have a wavy profile and stack in the radial direction. In both hoof and horn, the lamellae are composed of IFs, as in other α-keratin-based tissues (Figure 5). At a macroscopic scale, the horn has spiral ridges, which correspond to seasonal growth phases, and, in the live animal, it encloses a porous bone; the horn weight can span from 80 to 500 g according to age [23,40]. A tightly packed structure is at the basis of the mechanical resistance of hooves and horns; in particular, horns have a specific fracture load greater than many other biological and synthetic materials: 32 kJ/m^2^ against 6.6 kJ/m^2^ for antlers, 1.6 kJ/m^2^ for bovine femurs, 5 kJ/m^2^ for glass, and 26 kJ/m^2^ for mild steel [6,38]. 

Compared with other α-keratin tissues, such as wool and hair, horny keratins contain less cysteine, proline, threonine, and serine, and more glycine, leucine, tyrosine, and phenylalanine, as observed by Marshall and Gillespie [23] for sheep. In particular, the sulfur content in wool is 57% higher than that in hoof and horn, and these have a much more similar amino acid content to each other compared to wool [23]. Mineral elements are also found, although at a very low percentage and around 1%; potassium and sodium account for more than half of the total content [40].

#### 2.4.4. Pig Bristles

Pig bristles, also referred to as pig hairs or hog hairs, are one of the main pig by-products; they are non-edible, and they are not valorized in food applications like other non-edible pig by-products such as bones used for gelatine. Each pig generates ca. 0.9 kg of bristles [41,42]; in Europe, 110 million pigs are counted (according to the last available [39]), giving rise to ca. 99,000 tonnes of bristles each year. Very few studies are available on a deep physical–chemical analysis of bristles, and this is mainly because, until now, they have only been valorized at a “macroscopic” level, for brush production, and for fish and poultry meal [41,43]; no valorization at a molecular level, outside the laboratory scale, can be currently be found. 

Bristles are made up of keratin, the content of which is up to 90–95% [41,44]; they have a similar structure to wool and human hair and are based on α-keratin [44]. Measured against wool, bristles show greater amounts of glycine, proline, and serine and lower amounts of valine and alanine (Table 2). From a mechanical point of view, they show a similar behavior to that of wool [44].

## 3. Characterization of Keratin and Keratinous Tissue Structure

**Figure 6:**
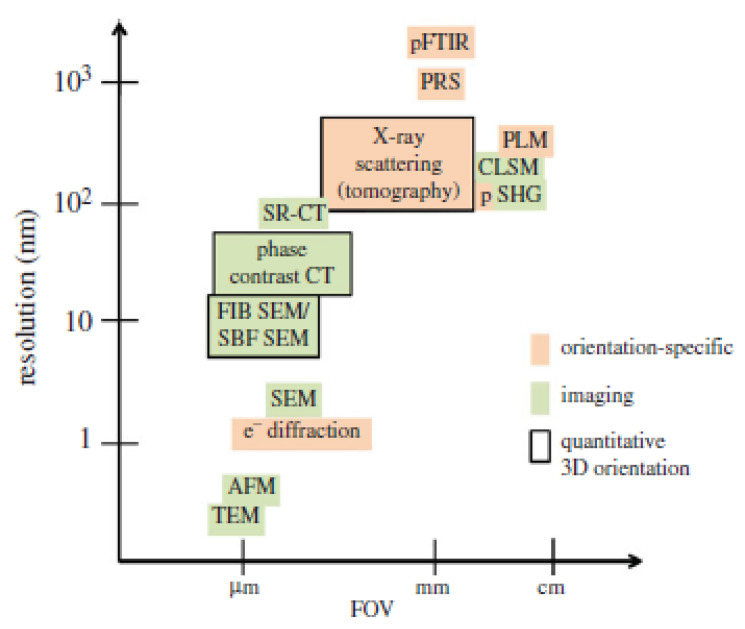
Imaging and orientation-specific techniques that can be applied for the characterization of materials like keratin and keratinous tissue structure. pFTIR: polarized Fourier transform infrared spectroscopy; PRS: polarized Raman spectroscopy; PLM: polarized light microscopy; CLSM: confocal laser scanning microscopy; SHS: second-harmonic generation microscopy; SR-CT: submicrometric range computed tomography; SEM: scanning emission microscopy; FIB: focused ion beam; SBF: serial block face; TEM: transmission electron microscopy; AFM: atomic force microscopy; FOV: field of view) (image from [45] with permission).

The structure of keratin and keratinous tissues can be characterized by several physical techniques, and these can be grouped according to the information provided (Figure 6 as an example) [45]. Spectroscopic techniques (like Fourier transform infrared (FTIR), Raman, terahertz (THz), solid-state nuclear magnetic resonance (NMR)) and X-ray-based techniques (like crystallography, small-angle X-ray scattering (SAXS), wide-angle X-ray scattering (WAXS)) give useful information about keratin conformation (α-helix, β-sheet, β-turns, random coils, etc.) and about the chemical compositions of keratinous tissues (presence of groups other than proteins, such as lipids, carbohydrates, minerals, and water) [45,46,47]. Imaging techniques based on electrons (such as scanning electron microscopy (SEM) and transmission electron microscopy (TEM)), photons (such as second-harmonic generation (SHS) microscopy), X-ray diffraction (such as micro-tomography), etc., can provide information about the orientation of keratin fibers and lamellae and about the assembly of the different constituents of the keratinous tissues [45,48,49,50]. In addition, spectroscopic and imaging techniques can be coupled (such as SAXS–tomography, magnetic resonance imaging (MRI), and SEM-EDXS (energy-dispersive X-ray spectroscopy)) to increase the field of view (FOV) of sole microscopy, which is lower than that of spectroscopy (Figure 5) [45]. 

Thermal analysis and calorimetry can be used to obtain information about the physical properties of keratin and keratinous tissues. Specific heat, glass transition, melting point, unfolding temperature, changes in *Gibbs* free energy, etc., can be determined with differential scanning calorimetry (DSC) [51,52], while weight changes (loss or gain) and rates of weight changes as a function of temperature, time, and atmosphere can be obtained by thermogravimetric analysis (TGA) [53]. 

In the following paragraphs, some studies on keratin and keratinous tissues using the above-mentioned techniques are reviewed in detail.

### 3.1. Spectroscopy Techniques

#### 3.1.1. Fourier Transform Infrared Spectroscopy (FTIR) 

FTIR spectroscopy is one of the most common and well-known methods for the study of the conformation of proteins and polypeptides through the analysis of their secondary structure (i.e., the content of α-helix, β-sheets, β-turns, random coils, etc.). In addition, by this non-destructive technique, proteins’ and peptides’ structural dynamics and stability, aggregation, and conformational changes under several conditions (e.g., ligand binding, temperature, pH, pressure) can be followed, and molecules other than proteins can be identified [54,55]. In the IR spectrum (4000–500 cm^−1^ frequency range for the most common equipment), protein bonds show several vibrational frequencies. Nine ranges are associated with their polypeptide chains: Amide A (3300 cm^−1^ approximate frequency, corresponding to the N-H bond stretching), Amide B (3100 cm^−1^ approximate frequency, N-H stretching), Amide I (ca. 1690–1600 cm^−1^, C=O stretching), Amide II (ca. 1580–1480 cm^−1^, C-N stretching, N-H bending), Amide III (ca. 1300–1230 cm^−1^, C-N stretching, N-H bending), Amide IV (ca. 770–630 cm^−1^, O=C-N bending), Amide V (ca. 800–640 cm^−1^, out-of-plane N-H bending), Amide VI (ca. 600–540 cm^−1^, out-of-plane C=O bending), Amide VII (ca. 200 cm^−1^, skeletal torsion) [54,55]. Among all these bands, the Amide I region is the most significant since it is directly related to the protein backbone structure; information on the secondary structure components can be obtained through the deconvolution or, more easily, through the second derivative of the Amide I band [55,56]. Protein secondary structure can be quantitatively estimated using the assumption that each protein is a linear sum of fundamental secondary structural elements, with the percentage of each element determined by the spectral intensity [54,55]. By comparing the high-resolution X-ray crystal structure of the protein with IR spectra, it has been possible to assign the 1658–1650 cm^−1^ frequency to the α-helix, 1640–1620 cm^−1^ and 1696–1690 cm^−1^ to the β-sheet, 1648–1640 cm^−1^ to the random structures, and 1670–1685 cm-1 to the β-turns [54,55,56,57,58]. 

Attenuated total reflectance (ATR) and transmission are the most common techniques for FTIR spectra acquisition. However, for solid samples like feathers, ATR is the most widely used thanks to its rapidity, ease of use, and high resolution [54,55,56,57,58,59]. The microstructure of raw wool (fleece of fine quality) and of keratin extracted from wool was investigated by Cardamone [27]. Contents of ca. 58% α-helices, ca. 38% β-sheets, and ca. 4% of unordered structures were found. These proportions were different after wool treatment by reducing agents, where ca. 37% α-helices, ca. 50% β-sheet, and ca. 13% disordered structures were noticed, and after alkaline hydrolysis, ca. 26% α-helix, ca. 52% β-sheet, ca. 22.5% disordered. These changes are due to the fact that, by reduction and alkaline hydrolysis, a change in the -OH, -NH, and -CO protein regions occurs [27]. In a study performed by Lin et al. [58], a comparison of the protein secondary structure in the shaft of several avian flight feathers was carried out. It was noticed that there was a higher amount of β-sheets in the cortex and a higher amount of α-helices in the medulla of the shaft. In addition, feather shafts have been shown to include a considerable amount of collagen (11–16% in the cortex, 13–16 % in the medulla). The pig bristle secondary structure was investigated by Mohan et al. [44]; it was observed to have a content of ca. 34% α-helices, ca. 37% β-sheets, ca. 15% β-turns, and ca. 18% disordered structures. The authors also noticed that heating at 80 °C significantly increased the content of disordered structures (up to ca. 28%), while heating up to 120 °C allowed a decrease in the content of the same structures (down to ca. 6%). 

Eventually, FTIR data can be analyzed through chemometrics, for instance, by principal component analysis (PCA) and hierarchical cluster analysis (HCA), to investigate similarities among different keratin sources and to estimate the effect of treatments on the changes in the original structure of keratin [47,54,57].

#### 3.1.2. Terahertz Spectroscopy

Terahertz (THz) spectroscopy is a very recent technique; it was discovered in 1989 [60]. THz radiation represents a large portion of the electromagnetic spectrum; it lies in the frequency range of 0.1–10 THz (3.33–333 cm^−1^ in wavenumbers, or 30–3000 μm in wavelength), i.e., between the microwave and infrared regions [60,61]. This spectroscopy can be very useful for the analysis of polar media, which absorb THz waves due to their intermolecular activities. Inter- and intramolecular vibrational modes of biological macromolecules such as hydrogen bonds, *Van-der-Waal* forces, and dipole rotational and vibrational transitions lie in the 0.1–10 THz frequency band. An important advantage of terahertz waves (and in particular of the sub-terahertz frequency range 0.1 THz–0.3 THz) is that many materials that block visible and IR spectra appear to be transparent in the terahertz region; thus, this radiation can well penetrate non-metallic, nonpolar, and dry materials (for example, wood, clothing, plastic, cardboard, ceramics) up to several millimeters [60,61]. In addition, electromagnetic spectrum frequencies in the range of 0.1–1.5 THz (3–50 cm^−1^) can be measured by terahertz time-domain spectroscopy (THz-TDS) by which the optical properties of materials, such as the absorption coefficient and refractive index, can be obtained [60]. 

Very few studies are available on the application of THz spectroscopy to proteins. This class of molecules does not show any specific spectra below 100 cm^−1^ wavenumbers. The first studies carried out [51] noticed a signal related to the hydration of the protein and polypeptides. Further research detected N-H-O and C-N torsional vibration correlated to the peptide backbone (Amide VII region) at the frequency of 6.0 THz [62]. In a study performed by Molloy and Naftaly [63], terahertz spectroscopy was used for the identification of wool in textiles of different animal origins. Several textiles, both woven and knitted, were analyzed in the range of 0.2–3 THz; although differences among samples were noticed, no specific peaks related to keratin conformation and peptide bonds were investigated. Further studies will be likely available in the years to come.

### 3.2. Microscopy Techniques

#### 3.2.1. Scanning Electron Microscopy (SEM)

Scanning electron microscopy (SEM) is the most common and widely used electron microscopy technique; it allows for a nanometric investigation of a material surface, and also for elemental composition characterization when coupled with X-ray microanalysis [50]. The structure of feather shafts (rachis and calamus, cortex and medulla) and barbs, for instance, can be easily observed starting from a magnification of 30 fold (Figure 2 as example); however, to observe keratin fibers, either in feathers or in other tissues, a magnification of 10,000–30,000 fold can be required, and high-resolution SEM equipment can be needed [6]. SEM can be a very useful technique for evaluating the effect of treatments on the nanostructure of keratinous tissues and evaluating differences between native and extracted keratin morphology. Cardamone et al. [64], for instance, compared the morphology of untreated wool with wool-extracted keratin by using different alkaline treatments, and they noticed an assembly in the form of flakes instead of fibers. In another work performed by Welu et al. [65], the morphology of keratin extracted from chicken feathers was observed; in this case also, a different assembly from the native fibers was noticed, where keratin powders assembled as macroparticles with a round cross-section and many micropores.

#### 3.2.2. Transmission Electron Microscopy (TEM)

Transmission electron microscopy (TEM) is a non-destructive technique largely used in the characterization of the morphology, crystalline structure, and elemental features of samples through a resolution from the nano- to the microscale. In both TEM and SEM, the image is created through electrons; the main difference is that in SEM, the image is obtained by detecting scattered electrons, while in TEM it is obtained by detecting the electrons that have passed through the sample. In addition, the depth of field and the field of view of TEM are lower than those of SEM, meaning that a higher magnification is reached in TEM (more than 50 million times maximum magnification against 1–2 million times in SEM) [66,67]. These characteristics make TEM useful for the observation of keratin micro- and nanoparticles in engineered tissues, for instance [68], where a greater magnification can be required with respect to keratin-made tissues such as feathers and wool. In another study conducted by Wang and Meyers [69], TEM was demonstrated to be very useful for the study of the complex and multiple hierarchical assembly of the feather shaft, with the observation, inside the cortex cells, of macrofibrils 50–300 nm in diameter composed of keratin fibers 30–50 µm in diameter.

#### 3.2.3. Second-Harmonic Generation (SHG) Microscopy 

Second-harmonic generation (SHG) microscopy is a form of multi-photon microscopy based on the use of a short-pulsed laser for the generation of a high spatial and temporal photon density in the sample to be observed; this is a quite recent technique, and its application has increased only over the past two decades [45,49]. SHG is label-free and can provide three-dimensional images with high resolution, thanks to the higher penetration depth in a tissue, the greater selectivity, and the higher photon yield [45,49]. In addition, SHG can produce a 3D contrast without the need to excite fluorescent molecules, which has multiple advantages such as preventing phototoxicity or photobleaching of the samples [70,71,72]. For its features, SHG has been shown to be very useful for bio-imaging, where morphological and biochemical information from biological tissues, such as bone and skin, and biological macromolecules such as proteins, polysaccharides, and fats, either in health or pathologic conditions, is investigated [45,49]. In the research work of Chen et al. [73], second-harmonic generation microscopy was applied to the study of human skin, which contains intrinsic materials such as keratin, collagen, and elastin.

### 3.3. X-ray-Based Techniques

#### 3.3.1. X-ray Diffraction (XRD)

X-ray diffraction is a phenomenon in which an incident monochromatic X-ray beam receives interference from the atoms of a crystal and is reflected at the same θ angle as the incident beam [74]. XRD can give information about structure, phases, crystal orientation, crystal size, and defects in either organic or inorganic materials. For instance, Li et al. [75] applied XRD to the characterization of wool keratin/hydroxyapatite nanocomposites; in their research, a series of nanocomposites were prepared by varying the concentration of keratin and calcium phosphate, and by XRD, it was observed that there was a decrease in the crystallinity of pure hydroxyapatite with an increase in the content of keratin. Al-Ashwal et al. [76] studied dehydrated keratin extracted from chicken feathers; the results of XRD analysis revealed that, after the applied drying conditions, keratin assembled in a semi-crystalline form, and two dominant peaks were present in the X-ray spectra, corresponding to α-helices and β-sheets.

#### 3.3.2. Small-Angle/Wide-Angle X-ray Scattering

Small-angle X-ray scattering (SAXS) and wide-angle X-ray scattering (WAXS) occur when a specimen scatters an incident X-ray beam in small (0.1–1°) and large angles (5–60°), respectively [45]. These high-resolution techniques have the advantage of allowing the study of biological samples in their original form and natural environment. WAXS can provide information at the sub-nanometric resolution, while SAXS can give information of a greater size, 1–100 nm. By SAXS, it is possible to study molecular mass, size distribution, shape, pore size, characteristic distances of ordered or partially ordered materials, quaternary structure, etc. In the case of biological materials, SAXS has an advantage over X-ray crystallography (also based on X-ray scattering) since a crystalline sample is not needed. The WAXS region, on the contrary, carries information about the secondary structure and how it changes under different stimuli and is widely used to study the crystallinity degree of polymer samples [77,78]. Saranathan et al. [79] applied SAXS to the study of the nanostructure, the refractive index, and the reflectance of 297 colored feathers from 230 different avian species belonging to 51 avian families. They noticed isotropic optical properties and a nanostructure assembly of keratin fibers in the form of channels and spheres, which are responsible for the slate-grey and blue-grey structural coloration of feathers.

#### 3.3.3. Micro-Computed Tomography (μCT) 

Micro-computed Tomography is an X-ray tomography technique with a sub-micrometer resolution. It uses X-rays to create cross-sections of objects/materials, which are assembled afterward by computer software to generate a virtual three-dimensional image [80,81]. By µCT, information on volume, shape, and size distribution of the inner micrometric structure of specimens can be obtained; in addition, stereological errors with respect to common bi-dimensional imaging techniques such as SEM can be reduced [80,81]. µCT also has the advantage of being non-destructive; however, the scanning time of one sample is much longer than for other imaging techniques, and can take up to 10 h; also, a large number of cross-sections are needed (in the order of thousands). Laurent and co-workers [82] applied µCT for the study of nano-mechanical properties of bird feather rachises; it was possible to show the anisotropy of keratin fiber orientation and the presence in the rachis of three distinct cross-section layers. In another study by the same author et al. [83], micro-computed tomography was used to observe laminar geometry in the shaft cortex of the feather, where in total, 4000 cross-sections were taken.

### 3.4. Thermal Analysis and Calorimetry

#### 3.4.1. Differential Scanning Calorimetry (DSC)

Differential scanning calorimetry (DSC) is a thermodynamic technique that measures the heat quantity change that is either radiated or absorbed from a sample as a function of temperature and time [51,52]. In the case of proteins like keratin, DSC allows measuring and determining several parameters, such as phase transition, melting point, crystallization kinetics, thermal and oxidative stability, folding, interactions, and thermodynamic entities (enthalpy, entropy, *Gibbs* energy) [84]. de Castro Lima and co-workers [85] reviewed the dominant literature on the DSC application in hair studies and in animal tissues such as wool and feathers, and they reported that only small differences were noticed between the keratin samples considered. A common result of all the reviewed studies was the identification of the temperature range 167–197 °C as the beginning of keratin degradation, with the formation of ammonia and CO_2_, followed by the generation of inorganic sulfur-containing compounds such as H_2_S and SO_2_, at 240–248 °C and 253–260 °C, respectively. Thiols are formed in two stages, 257 and 320 °C; nitriles, in the range 340–480 °C; and phenols, the most important degradation compounds, are formed in the range 370–400 °C. Cao et al. [86], for instance, reported on the melting behavior of dry and wet wool; they noticed a very low melting enthalpy (ca. 10 J/g) and a thermal degradation temperature of 185–198 °C depending on the heating rate. Through another DSC study, the same author [87] was able to find that human hair has very similar thermal characteristics to wool, yet its degree of crystallinity is higher when compared, for instance, to Merino wool.

#### 3.4.2. Thermogravimetric Analysis (TGA) 

Thermogravimetric analysis (TGA) is a technique in which the mass of a specimen is measured as a function of temperature and time while the sample itself is submitted to a controlled temperature program and in a controlled atmosphere. By mass changes measured through TGA, it is possible to characterize a material’s composition and predict its thermal stability [88,89]. Mass changes, in fact, occur while a sample loses or gains mass in different ways that originate from reactions inside the sample itself or reactions of the sample within the surrounding environment [53]. Ramakrishnan et al. [90] characterized chicken feather keratin bioplastic films with several concentrations of glycerol as a plasticizer agent. TGA showed that, whatever the plasticizer concentration, the keratin-based bioplastics had the same weight loss trend; however, an increase in glycerol concentration resulted in a significant increase in the thermal degradation over time, at a constant temperature.

### 3.5. Nuclear Magnetic Resonance (NMR) Spectroscopy 

NMR is a non-destructive technique that can be applied for the analysis of protein structure, functions, and interactions, at the atomic resolution; it can be used for solid- and liquid-state samples, either in vivo or outside a living organism [91,92]. Protein folding and function can be studied by NMR, and the advantage is that the analysis is less dependent on sample conditions compared to X-ray and electron microscopy techniques. However, for complex protein systems such as large protein complexes, disordered proteins, and multi-conformation proteins, NMR alone may not be sufficient, and a combination with previously discussed techniques is required [91,92]. In the case of keratinous tissues, as well as solid and semi-solid keratin-containing products, secondary structures such as α-helices, β-sheets, random coils, and amorphous regions can be visualized through solid-state NMR (ssNMR) [93,94,95,96]. Spectra from hair, hooves, and feathers have been compared and assigned using ssNMR; it was predicted that the secondary structure of keratin in those tissues is significantly heterogeneous, with α-helical rod domains coexisting with β-sheets and the presence of random coils and turn structures in the matrix proteins and in the keratin head and tail domains [97]. Sharma et al. [98] characterized keratin microparticles extracted from feathers; hydrolyzed feather spectra were recorded at 125 MHz with carbon-13 NMR. The NMR analysis showed a peak with a maximum between 38 to 43 ppm corresponding to β-carbons of leucine and cystine residues; peaks at 30–40 nm were assigned to glutamine, proline, and glutamic acid residues, and peaks from 10 to 35 ppm were assigned to the side chain of aliphatic carbons. Fagbemi et al. [99] used NMR in the optimization of keratin extraction from chicken feathers, through the characterization of keratin hydrolysates with solid-state nucleic magnetic resonance ^13^C NMR. These authors also noticed β-carbons at a shift of ca. 42 ppm and α-carbons at 60 ppm; aromatic carbons were seen between 120 and 150 ppm.

## 4. Methods for Extraction of Keratin from Livestock By-Products

The proteins of cornified tissue can be extracted by using a variety of solvents and denaturing agents; however, keratin is the third most difficult polymer to degrade/extract from the natural world after cellulose and chitin [14]. Keratin, in fact, behaves differently from other proteins, and conventional methods for solubilizing proteins typically fail to solubilize keratin due to the presence of disulfide (-S-S) bridges in particular. Yet, under controlled circumstances, and particularly under low pH and in the presence of reducing/oxidizing agents [8,100], the S-S, amino (-NH_2_), and carboxylic acid (-COOH) moieties can become more water-soluble and chemically reactive. In the following paragraphs, the up-to-date known methods are reviewed, and they are summarized in Table 4.

### 4.1. Chemical Methods

#### 4.1.1. Acidic Hydrolysis

In acidic methods, livestock keratinous tissues are immersed in a strong acidic solution to undergo hydrolysis; after a given duration, the solution is neutralized and more often dried to stabilize the final product. Strong acids like HCl and H_2_SO_4_ are generally needed [1,2,4,15]. An increase in the time of hydrolysis can increase the yield of the extracted keratin; however, it will result in lower-molecular-weight compounds; hence, the appropriate duration should be chosen with respect to the envisaged functionalities and application [1,15]. Acidic hydrolysis is an efficient process for keratin extraction, but the nutritional value of the peptides obtained is low, and some amino acids like tryptophan may be lost. The main disadvantage of acidic treatment is the high amount of strongly acidic waste produced, which can expose equipment and the environment to possible dangers [101].

#### 4.1.2. Alkaline Hydrolysis

Alkaline hydrolysis of keratinous tissues is more often carried out with hydroxyl compounds like Ca(OH)_2_, KOH, and NaOH. During alkali extraction, decarboxylation, desulfuration, and deamination can also occur, however, unlike the acidic treatment, all the amino acids of the original protein are preserved [1,2,4]. Nevertheless, the alkali treatment generally results in a loss of the mechanical properties of keratin and in a brown color; also, the fraction with lower molecular weight cannot exhibit film-forming properties [102]. Cystine residues were reported to be the most affected sites of the protein chain [102], and it has also been observed that a smaller cationic radius of the alkali is more effective than the strength of the alkali itself; for instance, LiOH was more effective than KOH, and, in turn, the latter was more effective than NaOH when applied to wool hydrolysis (5 g of wool in 20 mL 0.2 N alkali) [102]. In many cases, a combined acidic–alkali treatment has been observed to be more efficient than one of the two alone [1,4,99,101].

#### 4.1.3. Oxidation

The first studies on keratin extraction by oxidation date back to 1950, by Alexander and Earland [103], after which several studies on the topic were carried out. The oxidation is mainly carried out by performic acid, peracetic acid, and hydrogen peroxide, which are able to break down the disulfide covalent bonds into residues of cysteic acid. The extracted keratins, named keratoses, have a structure crosslinked by non-covalent bonds; these keratins are hygroscopic and water-soluble [2,4,103]. Keratoses show different behaviors according to the pH and are classified as follows: α-keratoses, which are extracted from the cortex, have a crystalline structure and are isolated by solubilization in ammonia followed by precipitation at pH 4; β-keratoses, which derive from the cell membrane and from the cuticle and are insoluble in ammonia; γ-keratoses, which are soluble in ammonia but do not precipitate in acidic conditions [4]. The ratio of α/β/γ-keratoses can be different according to the tissues; for instance, it was reported as 60/10/30% in wool and 31/18/35% for feather barbs [4]. De Guzman et al. [104] used 2% peracetic acid at 37 °C and for 10 h to extract keratoses from human hair. During the extraction procedure, disulfide covalent bonds were disrupted to generate cystic acid and sulfonate groups, and cysteine dioxide and monoxide were produced as well. Although α-keratin is more soluble than β-keratin, the oxidation process often removes more of it. Some amino acids, including tryptophan, methionine, cysteine, serine, threonine, tyrosine, histidine, and phenylalanine, can be destroyed by strong oxidative substances [105,106]. In addition, long extraction times (up to 3 days) and high acid concentrations (for instance, up to 36% of peracetic acid) can be required to obtain > 50% recovery yield [107].

#### 4.1.4. Reduction

Reductive extraction of keratin is generally carried out by aqueous solutions of reducing agents such as 2-mercaptoethanol and/or thiols (dithiothreitol, sodium thioglycolate, thioglycolic acid, etc.) able to break disulfide bonds. Denaturing agents such as urea at high concentrations (up to 8 M) and surfactant compounds such as sodium hydrogen sulfite, sodium sulfate, and sodium dodecyl sulfate (SDS) are often used in combination with the reducing agents [4,107,108,109]. Protein denaturants can break down the hydrogen bonds and change the structure of extracted keratin, which results in an increase in the dissolution capacity of the reductant, and surfactants can increase the stability of the extracted keratins [107,108]. Reduction is the most widely used chemical method for the extraction of both α- and β-keratins; however, the use of 2-mercaptoethanol and thiols has high costs and toxic effects, and agents like sodium disulfite are preferred since they are cheaper and less toxic even if lower yields are obtained [4,107,108,109]. For instance, Zhou et al. [109] reported that the use of 0.125 M Na_2_S_2_O_5_, 0.05 M SDS, and 2 M urea is an efficient way to break disulfide bonds in wool without structural damage to the keratin tertiary structure and with a dissolution yield of ca. 49%. The obtained keratin hydrolysates of ca. 14.4 kDa molecular weight were able to self-assemble in the form of sponges. Keratin extraction by reducing agents has also been applied to other livestock sources of keratin, such as feathers, horns, and hooves, although these sources contain less cysteine compared to wool, and the extraction yield is significantly lower [4].

#### 4.1.5. Ionic Liquids (ILs)

ILs are liquids that exist only in ionic form (either anions or cations); they are also represented by salts that exist as liquids below 100 °C or at room temperature [1,110,111]. The solvation capacity of these liquids is higher compared to traditional solvents, and they have a strong potential for application in the extraction of natural polymers such as keratin. ILs have high melting and boiling points and are categorized as eco-friendly, non-volatile, non-flammable, non-corrosive solvents with respect to many acids, minerals, and alkalis; ILs also have high thermal and chemical stability and are associated with specific chemical, physical, and biological properties. The strong polarity of ILs can break the intermolecular bonds of livestock keratin, such as hydrogen bonds, resulting in an increase in the dissolution yield [101,111]. However, to facilitate keratin dissolution in ILs, the disulfide bonds of cysteine should be disrupted, and this is why co-solvents such as Na_2_SO_3_, NAHSO_3_, SDS, and urea are added to increase yields and reduce durations. Xie et al. [111] tested several ionic liquids, including BMIM^+^Cl^−^ (1-butyl-3-methyllimidazolium chloride), Br^−^, BF_4_^−^, PF_6_^−^, and 1-allyl-3-methylimidazolium chloride (AMIMCl), to break hydrogen bonds in wool keratin. High temperatures were used, 100–130 °C, for 10–24 h. The solubility yields were low (up to 11%), yet the extracted keratin was more thermally stable than the natural wool keratin fibers. A more recent study [112] reported the use of ILs to extract keratin from chicken feathers with yields up to 75% by combining imidazole ionic liquids with Na_2_SO_3_. It was also possible to reduce the extraction temperature (90 °C) and time (60 min) with respect to previous studies on wool.

### 4.2. Biological Methods

Biological methods can have many advantages over chemical extractions since they require less energy, avoid the destruction of several amino acids, and require mild conditions besides avoiding some forms of environmental damage. Biological extraction can be carried out either with microorganisms or with extracted or synthesized enzymes. The yields of microbial and enzymatic keratin recovery were found comparable in many cases and higher than those of chemical methods since two steps concomitantly occur, i.e., reduction of disulfide bonds and cleavage of the peptide bonds. However, the molecular weight of the keratin fraction recovered can be lower, and the application is different from chemical methods; in addition, the price of enzymes and microorganisms can be higher compared to chemical compounds [4,113,114].

#### 4.2.1. Microbial Methods

Microorganisms utilized to extract keratin have been mainly isolated from soil and poultry waste, for which the *Bacillus* genus is the most abundant; in this genus, *Bacillus licheniformis* was demonstrated to be the most performant [4,113,114]. Apart from *Bacillus*, *Amycolatopsis Chryseobacterium*, *Streptomyces, Staphylococcus,* etc., are common keratin-degrading bacteria. Fungi, in particular, thermotolerant ones such as *Chrysosporium tropicum*, *Aphanoascus fulvesence*, and *Microsporum gypseum*, were also demonstrated to be performant; however, the mesophilic fungi represent the majority of fungi having keratinolytic activity [15,115]. In the majority of cases, bacteria and fungi are coupled, and denaturing agents such as urea are added to increase yields and target a specific molecular weight [4]. Depending on their keratinolytic activity, microorganisms can be categorized into two groups: (i) strong proteolytic activity with a direct action on keratin hydrolysis and solubilization, (ii) potential keratinolytic activity with a direct action on the non-keratin protein matrix and the release of keratin itself from the network [116].

#### 4.2.2. Enzymatic Hydrolysis

Keratinases are the most effective enzymes for keratin extraction and they are commonly found in bacteria, actinomycetes, and keratinophilic fungi; on the contrary, common proteases such as pepsin and trypsin cannot hydrolyze livestock keratin [115,116,117]. Keratinases can be located inside the cell, in the membrane, or outside the cells of the microorganism, although, during the process of metabolism, they are released in the microorganism environment, facilitating purification [115,116]. The *Apergillus*, *Lysobacter*, *Bacillus,* and *Streptomyces* genera contain the highest amounts of keratinases [115,116,117]. As for microbial extraction, also when using keratinases, a beneficial effect of using surfactant and denaturing agents was observed in the case of wool and feather treatment [118]. Keratinases are active from very low to very high pH and temperatures, where very high or very low pH with prolonged exposure at elevated temperatures results in low-molecular-weight peptides and free amino acids, useful for application in fertilization and animal feed [117,118] for instance. Su et al. [119] studied the release of high-molecular-weight keratin fractions from wool, in the range of 10–45 kDa, which, on the contrary, are demanded for biomedical applications, such as injectable hydrogels for wound healing and possible drug delivery.

### 4.3. Novel Methods

A number of novel techniques have been applied to the recovery of keratin from several tissues. Deep eutectic solvents, ultrasounds, pulsed electric fields, high pressures, microwave-assisted extraction, and steam flash explosions have been tested with the aim of improving the keratin extraction yield and functionality. However, these techniques also have some drawbacks, such as a high price or complex mechanisms of action; hence, more research is needed, and expected in the near future, to improve keratin extraction techniques [120,121,122]. 

Apart from keratin extraction, novel methods can be useful for the synthesis of keratin-based livestock products. For instance, Zuliani et al. [43] studied the valorization of pig bristles with microwave-assisted extraction for the production of a high-performance photocatalyst, *pb*-Cu_2_S (*pb* means “bristles derived”). A microwave power of 500 W, 200 °C, was applied to make pig bristles react with CuCl; bristles started decomposing upon irradiation, the disulfide bridges opened, and copper sulfide was then formed by S_2_^−^ substitution of Cl^−^. The photocatalyst material produced in this research was demonstrated useful for the degradation of methyl red, which opens the door to other possible utilizations.

**Table 4:** Methods for extraction of keratin from animal by-products.

Keratin Source	Extraction Technique	Extraction Conditions	Product/Application	Reference
Mixture of keratinous tissues	Hydrothermal pre-treatment followed by microbial hydrolysis with fungi	50–55% humidity, 170–180 °C, 60 s pre-treatment; 4 g of pre-treated material incubated with keratinase extracted from *Acremonium chrysogenium*, ratio E:S 1:4, 1–8, in 1 L reactor, 55 °C with continuous stirring, for 1–6 h	Low-molecular-weight keratin hydrolysates and free amino acids with 100% bioavailability	[123]
Chicken feathers	Reducing agent	Chicken feathers treated with calcium hydroxide; temperature of 50–150 °C, duration of 0–300 min, and varying raw material concentration followed by centrifuge separation	The final product is rich in soluble amino acids and polypeptides and can be used for animal feed; it can be a potential protein source for ruminants	[124]
	Alkaline enzymatic hydrolysis	Pre-heating of ground chicken feathers by boiling water, followed by addition of lipolytic enzymes and pH adjustment; 24 h stirring followed by the addition of 0.3% *w*/*w* KOH in water for alkaline hydrolysis; separation through filtration	Preparation of water emulsions for cosmetic application, containing 2, 4, and 6% by weight of keratin hydrolysates for dermal use	[125]
	Alkaline hydrolysis	Defatted and milled chicken feathers into NaOH aqueous solution at different concentrations in a wide range of pH, temperature, and reaction time	Purified keratin hydrolysates with biochemical properties; bio-adhesives	[99]
	Alkaline extraction	Washed, dried, and defatted chicken feathers by soaking in ether for 24 h; extraction of keratin from 50 g of feathers in 1 L of 1 M NaOH for 24 h, followed by stirring for 5 h at 50 °C; centrifugation at 10,000 rpm to remove biomass waste	Nitrogen source and pH regulator for microbial culture in the production of lactic acid bacteria from date pulp waste by fermentation	[126]
	Reduction by L-cysteine	Cleaned chicken feathers in 8 M urea and L-cysteine, 1:17 liquor ratio; pH 10.5 using 50% *w*/*w* NaOH; 12 h stirring at 70 °C; 10,000 rpm, 20 min centrifugation and filtration	Keratin fibers with potential application in the biomedical field for tissue engineering and drug delivery; high yield or recovery, 60%	[127]
	Enzymatic digestion with keratinase	Cibenza IND900 keratinase from *B. licheniformis*, 1 g in 30 mL phosphate buffer saline, pH 9; 1:30 to 1:30,000 dilution tested; 1 g feathers added to 3 mL of each dilution, 45 °C, 12 h reaction time	Recovery of glucocorticoids from feathers and other non-protein analytes from keratinous tissues	[128]
Duck feathers	Ionic liquid	Feathers immersed in 8 M urea, 4 mM 1,4–dithiothreitol, or 8 mM cysteine, 1:17 liquor ratio, pH 8, 70 °C, 12 h. Oven-drying and pulverization	Keratin filaments with increased ductility with respect to natural feathers; 60% extraction yield	[129]
	Imidazole ionic liquid	Different ratios of ionic liquid, feathers, Na_2_SO_3,_ and water; separation of solid keratin from liquid by filtration	Keratin hydrolysate with a dissolution rate of 96.7% and extraction yield of ca. 75%	[112]
Turkey feathers	Ionic liquids assisted by ultrasounds	0.5 g of cleaned feathers in 20 mL ionic liquid; sonication at 20 kHz and varying powers, 120, 200, and 280 W, 130 °C; followed by mechanically stirring until complete feather dissolution in the solvent	Biodegradable films and other applications in materials; increased thermal stability of regenerated keratin compared to raw feathers; higher yield of recovery and lower extraction times when compared to conventional methods	[130]
	Enzymatic treatment with alkaline keratinase produced from the *Aspergillus* sp. DHE7	20 g of feathers pre-treated with 1 L dimethyl-sulfoxide, heated at 100 °C for 2 h; 8000 g, 10 min, 4 °C centrifugation to collect the precipitate; 1 mL enzyme solution to 1 mL keratin solution (1% in 50 mM Tris-HCl, pH 8); incubation at 50 °C for 30 min, reaction stopped with 15% trichloroacetic acid; centrifugation to collect the supernatant	Culture media for *Aspergillus* sp. DHE7, which has potential applications in laundry detergents, biocatalysts, production of keratin hydrolysates for feed use	[131]
Feather mix from poultry industry	Hydrolysis with microbial keratinases	Keratinase purification from *Bacillus* genus (*B. licheniformis*, *B. subtilis*, *B. pumilus*) and fungi (*Microsporum fulvum*, *Paecilomyces marquandii*). Complete solubilization of feathers achieved by incubation for 6 h, from 45 to 60 °C	Keratin-derived polypeptide chains that can be used to improve feed formulations, production of organic soil fertilizers and bioactive peptides with anti-hypertensive and antidiabetic capacity	[132]
Wool keratin	Reduction with L-cysteine	Wool fibers placed in a mixture of aqueous solution of 8 M urea and 0.165 M L-cysteine; pH adjusted at 10.5 with NaOH, followed by shaking at 75 °C for 5 h	Keratin powder with higher β-sheet, lower α-helix, and lower disordered structure contents than native wool	[28]
	Moderate hydrolysis by keratinase	Wool immersion in a water solution at pH 10 and stirred for 1 h at 65 °C, followed by addition of keratinase under continuous stirring at 50 °C for 48 h, centrifugation, and freeze-drying	Biomedical materials, accelerated wool healing	[118]
Sheep wool	Ionic liquids assisted by ultrasounds	Wool fibers washed in 1:1 *v*/*v* hexane and dichloromethane; 0.5 g of wool dried and cut into small pieces added to 10 mL of ionic liquids; ultrasonication at 130 kW, 50 Hz for 15 min; 4000 rpm, 15 min centrifugation at room temperature to collect the precipitate	High-molecular-weight keratin hydrolysates (37–75 kDa); innovative extraction technique with the potential for large-scale application	[133]
Merino wool	Multiple techniques (alkali hydrolysis, sulfitolysis, reduction, oxidation, ionic liquid)	*Reduction*: dried and defatted wool treated with urea and 2-mercaptoethanol; *Sulfitolysis*: wool treated with a mixture of urea and sodium metabisulfite; *Alkali hydrolysis*: wool treated with 2% *w*/*w* NaOH; *Oxidation*: wool oxidized with 2% *w*/*v* peracetic acid for 12 h at 25 °C; *Ionic liquid*: wool dissolution in 1-butyl-3-methylimidazolium chloride (BMIM)	Biomedical products without toxicity on fibroblast cells	[134]
	Ionic liquid	Wool fibers cleaned with ether, cut into small pieces, and immersed into ionic liquid at a ratio of 1:6 *w*/*w*, at 120, 150, and 180 °C for 30 min. Distillation of the hot mixture and precipitation of water-insoluble keratin; 4000 rpm, 15 min centrifugation to remove the ionic liquid	High-molecular-weight keratin hydrolysates (35–75 kDa) for the production of stretchable keratin films/sheets	[135]
	Thermal treatment and electrospinning	Cleaned and ether-defatted wool fibers, cut in millimeter pieces, treated with 8 M urea, 0.5 M Na_2_S_2_O_5_, pH 6.5 adjusted with 5 M NaOH, 1:20 liquor ratio, 2 h reaction time under shaking, 65 °C; filtration and dialysis against distilled water; freeze-drying to obtain pure keratin powder, followed by electrospinning	Keratin hydrolysates of 11–60 kDa molecular weight; pure keratin nanofibers; novel thermal stabilization to enhance thermal and water stability of the obtained pure keratin extract	[136]
Pig bristles	Enzymatic digestion and degradation by *B. cereus* (B5esz) under several conditions	Condition 1: thermo-chemical pre-treatment followed by enzymatic digestion; Condition 2: enzymatic digestion of untreated feathers in the presence of sulfite; Condition 3: thermo-chemical pre-treatment followed by microbial degradation	Solutions rich in branched amino acids. Biodegradation of bristles with *B*. *cereus* culture, instead of B5esz alone, resulted in a more complex peptide composition	[137]
	Two-step pre-treatment followed by microbial digestion with bacteria	Bristle cleaning; culture of *B. cereus* PMC2849 on cleaned bristles followed by hydrolysis of 10 g of pig bristles in 250 mL distilled water and 50 mL broth of keratinase extracted from *B. cereus* PCM 2849; autoclavation of the mixture in a sodium sulfite solution (1 g of bristles in 100 mL)	Free amino acid mixture rich in branched residues, for non-feed application	[138]
	Thermal pre-treatment followed by microbial digestion with fungi cultivated with a novel fermentation technique	Chopped and thermally pre-treated bristles at 150 °C, 600 kPa, 20 min, dried and cut into 1.4 mm size; two-stage fermentation process by *A. keratiniphila D2*, in the 28–88 °C temperature range and 5–11 pH range	Keratin small peptides and free amino acids with high pepsin digestibility, 95%, with potential application in animal feed; high yield of recovery, 73%	[139]
	Thermal hydrolysis	Two heating steps: (i) swelling and denaturation of the keratin network, (ii) cleavage of the disulfide bonds. 20 g of washed and dried hog hair in 1 L deionized water and in stirring conditions; 3°C/min heating rate up to vapor generation, then from 100 to 220 °C to break S-S bonds	High-molecular-weight keratin hydrolysates (20–100 kDa) and a wide range of weight distribution; high yield (ca. 70%) and comparable to chemical processes; compared to soybean meals, on dry matter, the extracted hydrolysates can provide twice as much essential amino acid content	[140]
Bovine hoofs	Reduction	Defatted hooves treated with 7 M urea, sodium lauryl sulfate, and 2-mercaptoethanol, under shaking for 12 h at 60 °C and pH 7	Production of a biocompatible material for cellular attachment and biomedical applications	[141]

## 5. Application of Keratin from Animal By-Products

As highlighted in the previous section, a wide range of keratin products can be obtained from animal by-products, and through different extraction methods. These products can have multiple applications, and the most promising ones are reviewed in the following paragraphs.

### 5.1. Bio-Based Plastics

**Figure 7:**
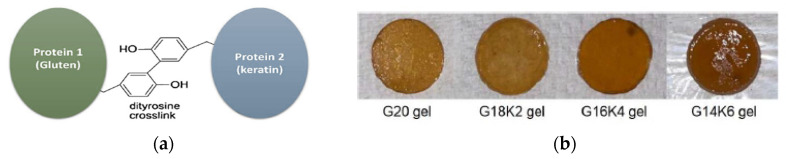
Chemical bonds formed by tyrosine photocrosslinking of keratin and gluten (**a**) and obtained keratin–gluten films at different gluten/keratin ratios (**b**) (image adapted from [144]).

Nowadays, plastics are used in a wide range of sectors, mainly as packaging for food, water, and drugs, and also for the fabrication of medical devices, automotive and communication technology components, textiles and geotextiles, etc. [142]. The greatest share of these plastics is of fossil origin, and the bio-based analogs represent less than 1% of the total 390 million metric tonnes of annual plastics production. Nonetheless, an increase is expected from 2.23 million tonnes in 2022 to 6.3 in 2027, also thanks to a strong development in new natural polymeric materials [143]. In this context, in the last 10 years, several studies have been carried out on the potential of keratin. Ramakrishnan et al. [90] studied the mechanical and thermal properties and the biodegradability of keratin films obtained from chicken feathers and by its extraction with sodium sulfide. The obtained protein was mixed with different proportions of glycerol (2, 5, and 10% by weight) as a plasticizer. The results were highly encouraging, mainly due to the lower concentration of glycerol. Fernández-d’Arlas [29] studied keratin plastics from wool; keratin fractions of 13, 22, and 31 kDa were solubilized by oxidation with H_2_O_2_ and NaClO, and glycerol and sodium dodecyl sulfonic acids were used as plasticizers, to impart different hydrophilicities. These films were transparent, able to absorb UV radiation, thermally stable up to 200 °C, and biodegradable by 40% after 5 days (where 100% degradation of the sole keratin, i.e., without plasticizer, is observed in the same conditions). Alshehhi et al. [144], recently formulated hybrid films based on keratin and gluten, plasticized by photocrosslinking (Figure 7). The advantage of blending different natural polymers is the modulation of several factors such as oxygen permeability, mechanical strength, thermal stability, and hydrophobicity, which have a strong influence on biodegradability. 

The authors showed that, with an increase in the keratin content (up to 30% *w*/*w*), the films’ hydrophilicity increases, along with the water uptake, the micropore size, and the biodegradability, while the viscoelasticity and the thermal stability decrease. Whatever the keratin content, all films exhibited good rheological properties allowing 3D (three-dimensional) printing. Keratin biodegradability is of great interest for the development of geotextiles; these products were applied for the first time in the 1980s and are defined as “permeable textiles used in conjunction with soil, foundation, rock, earth or any geotechnical engineering-related material” [145]. Vadillo et al. [146] addressed the biodegradability in soils of finely ground chicken feathers treated with a steam explosion, in order to evaluate their potential for geotextiles. The authors noticed that by modulating the severity of the steam explosion conditions, it was possible to modulate the biodegradability, and that the biodegradability was higher than that for the non-treated feathers; in particular, the biodegradability reached ca. 70% after 60 days, which was comparable with cellulose. Apart from keratinous animal by-products, human hair waste has also been studied for the production of bio-based films. Shubha et al. [147] formulated human hair keratin films of 190–220 μm thickness and used different types of plasticizers to assess mechanical properties and biodegradability using keratinolytic fungi. They noticed a film degradation of 80% after 7 days. Considering the current significant demand for bio-based plastics, it is expected that more studies on the keratin potential for this application will follow in the near future. 

### 5.2. Biomedical Domain 

**Figure 8:**
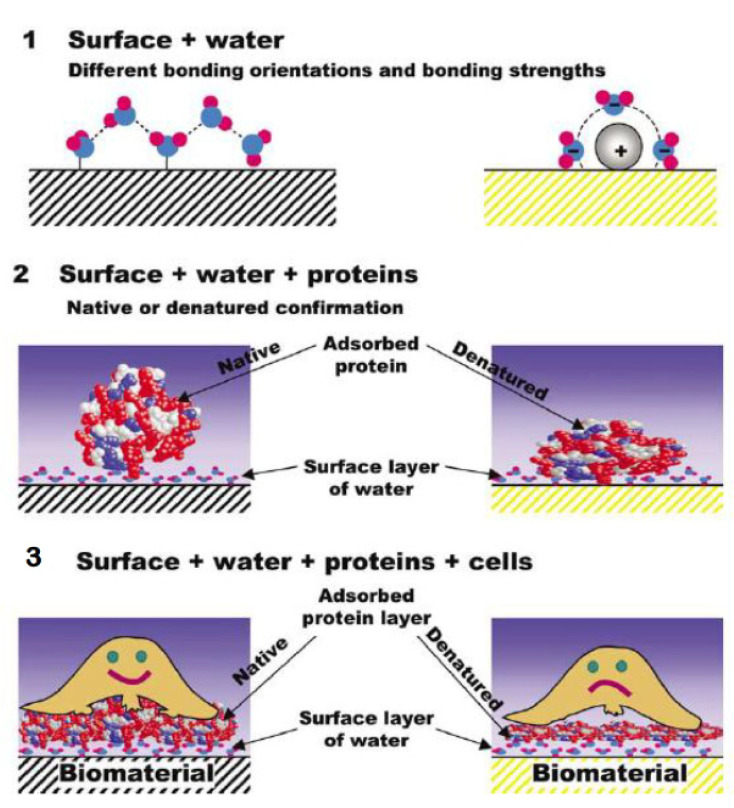
Action of a biomaterial after implantation in the human body: (**1**) accumulation of water and formation of an ion layer on its surface; (**2**) adsorption of proteins; (**3**) adhesion and spreading of cells (image adapted from [149] with permission).

The biomedical field is one of the most promising and demanding for keratin-based products. The application of keratin for medicinal purposes, and in particular as a biomaterial, is very ancient; the first documented uses date back to the 16th century in China; wool and hair were the first used tissues [9,25]. Biomaterials, which can be described as a synthetic extracellular matrix, must act as a scaffold and must simulate the structure and function of the native tissue (Figure 8); as such, they should promote cell proliferation and differentiation [9,148]. 

**Figure 9:**
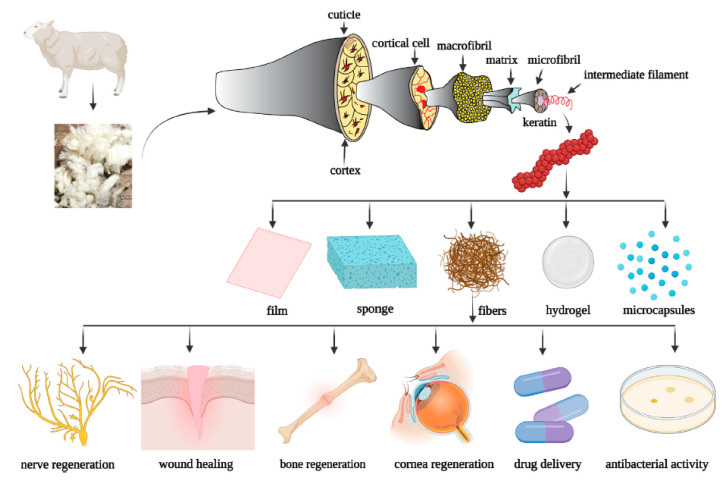
Different sheep wool keratin biomaterials and their target applications (image from [151] with permission).

**Figure 10:**
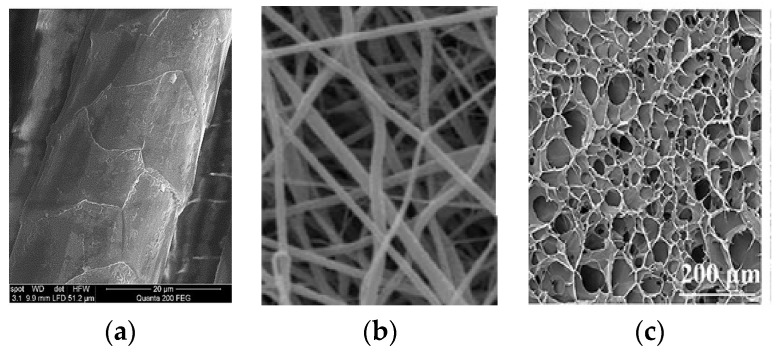
(**a**) SEM image of sheep wool fibers (image from [152]; magnification 5000×); (**b**) SEM of image nanofibers obtained from sheep wool (image from [153]; scale: 1 cm = 1.5 μm); (**c**) human hair keratin hydrogel (image from [154] with permission).

Keratin-based biomaterials have been the subject of extensive research in recent decades due to their inherent biological properties, exceptional biocompatibility, and mechanical durability [9,101]. Cell attachment and proliferation have been observed in several keratin scaffolds, sponges, films, and hydrogels in virtue of their self-assembly as nano- and macroscale networks, which can also retain higher amounts of water [148,150]. These biomaterials can be applied for nerve, bone, and cornea regeneration; wound healing; drug delivery; and antibacterial activity (Figure 9 and Figure 10). 

Keratin biomaterials produced, for instance, by solvent casting techniques, thermal processing, and electrospinning, starting from wool or hair, have been used as a carrier for bone morphogenic proteins, for the reconstruction of the ocular surface, for urinary tract engineering, and for nerve regeneration [148,150]. Authors de Guzman et al. [155], evaluated the efficacy of a keratose (i.e., oxidized keratin and the water-soluble keratin fraction) scaffold for bone regeneration, and compared it to the commercial product *Infuse*©, a crosslinked collagen scaffold, with respect to the release of bone morphogenic protein 2 (BMP-2) in the bone after implantation. The results showed the right delivery activity of BPM-2 by the keratose scaffold, leading to a good remodeling of the bone after fracture and with comparable efficacy with respect to the commercial collagen scaffold. Borrelli et al. [156] compared the efficacy of a keratin film from human hair for ocular surface reconstruction and compared its efficacy to the tissue commonly used for this purpose, i.e., the human amniotic membrane. Since this product has low biomedical strength and low transparency, alternatives are currently being studied. The results showed that cornea epithelial cells were able to grow on the keratin film, resulting in significant cornea healing; only small signs of inflammation were noticed. 

Keratin-based biomaterials have also shown the potential to deliver drugs and bioactive substances that can speed the regenerative process in wound healing or target specific body tissues and organs (blood, liver, skin, etc.) [9,25,101]. A drug delivery system is conceived to provide a therapeutic amount of a drug and to target the location, rate, and time of release of the drug itself in the body. Keratin nanoparticles have demonstrated efficacy in particular in the delivery of both hydrophobic and hydrophilic drugs. The delivery of molecules such as chlorhexidine (used to prevent skin and buccal mucosa infections); doxoburcine (used to treat malignant tumors); antimicrobial metal ions such as silver, copper, and zinc; and polysaccharide polymers, such as chitosan, was studied by several researchers, with promising observations [157]. 

**Figure 11:**
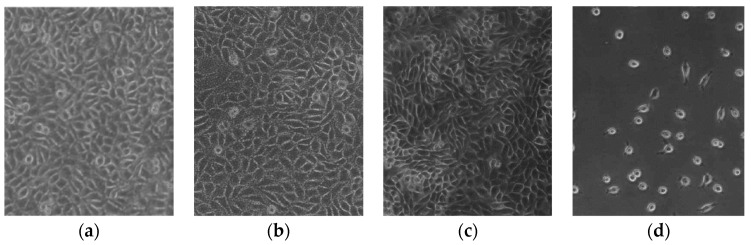
L929 mouse fibroblasts cultured on (**a**) keratin-coated dish, (**b**) keratin–chitosan (1:1 mix)-coated dish, (**c**) chitosan-coated dish, (**d**) untreated dish, at 96 h after seeding, at 37 °C (image adapted from [158] with permission).

In contrast to other natural polymers such as collagen, starch, and chitosan, the intricate three-dimensional structure of keratinous animal tissues necessitates the utilization of rigorous chemical and/or biological conditions for keratin dissolution and extraction. The selection of the right extraction process and pre-treatment is also of crucial importance for guaranteeing certain mechanical properties (some examples in Table 4) [9,101]. In fact, the main drawback of keratin-based biomaterials generally is the poor mechanical strength (like many other natural biomaterials); however, this can be overcome by the addition of natural compounds and other biopolymers able to increase the elasticity and plasticity, such as glycerol and sorbitol, or polysaccharides from cellulose and marine organisms. For instance, Tanabe et al. [158] extracted keratin from wool by reduction with urea, sodium dodecyl sulfate, and mercaptoethanol and formulated composite keratin films incorporating chitosan and glycerol. They noticed that the addition of 10–30% *w*/*w* of chitosan produced a stronger and more flexible product (27–34 MPa strength, 4–9% elongation) with respect to a pure keratin film. On the contrary, when chitosan concentration was less than 5% *w*/*w*, the film was too fragile to determine its mechanical properties. The addition of glycerol allowed an increase in the film elongation up to 24%; however, the strength was lower (13–14 MPa) with respect to the addition of chitosan. Apart from improving the mechanical properties, chitosan also allowed an increase in the antibacterial activity of the film towards *Escherichia coli*, from a 23% reduction rate for the sole keratin film to 62% with a keratin–chitosan formulation. Cell adhesion and proliferation studies showed that mouse fibroblasts were able to grow and converge on a keratin–chitosan (1:1)-coated dish, and that their morphology was more like that seen with keratin alone than that seen with chitosan alone (Figure 11). 

### 5.3. Biosorbents

Natural polymers like keratin have a strong potential for application in wastewater treatment thanks to their important sorbent capacity [159,160]. Toxic chemicals, heavy metals, dyes, petroleum derivatives, pathogenic microorganisms, etc., often need to be removed from industrial effluents, and with cost-effective solutions. In the last few decades, many studies have explored the potential of low-cost biosorbents, including keratin [159,160]. The biosorption process is based on a mass transfer from the polluted source to the surface or network structure of the natural sorbent, which must show good accumulation capacity [160]. Several mass transfer mechanisms have been hypothesized, and ion exchange has been noticed as the most important; the separation is based on a passive binding of organic and inorganic materials on the biosorbent’s polar active sites. A number of factors, however, can influence the adsorbate–biosorbent interaction, such as temperature, initial concentration, adsorbent quantity, contact time, and pH, which plays the most crucial role [1,160]. Keratin molecule chains contain ionic functional groups such as hydroxyl (R-OH), carboxyl (R-COOH), amino, and sulfhydryl groups (R-SH), which result in very good adsorption properties. Keratinous by-products, and hair, have been applied as biosorbents either without conversion or as keratin hydrolysates and engineered products in the form of sponges, fibers, and hydrogels. Keratin extracted from wool and chicken feathers was demonstrated to be a very good adsorbent for metals such as Pb (II) and Cd, Ni, Cr and Zn [101]. 

### 5.4. Biofertilizers

Modern agricultural systems are looking at improved technologies and products able to guarantee good production and environmental protection while reducing resource and energy use. In this context, keratin represents a promising biofertilizer; it is a very good source of essential nutrients for plant growth, such as carbon, nitrogen, sulfur, and some micronutrients. In addition, keratin fibers can retain water in the soil, contributing to plant hydration and water saving; they can improve the soil microbiome, and the keratin amino acids can stimulate microorganism activity on plant roots [161,162,163]. Wool has been by far the most used animal product for fertilization purposes; it gradually releases nitrogen into the soil thanks to the action of keratinases from soil bacteria; it can be very useful when a decrease in the pH is demanded, as in highly alkaline soils [161]. Over the past few decades, many efforts have been made to replace chemical fertilizers with bio-based ones, since synthetic compounds have significantly affected the environment and the natural microbial diversity in soil, reducing soil fertility and crop quality, in combination with prolonged use. Even though more research is needed on biofertilizer production, mechanisms of action, and performance for several plants, the available studies have highlighted, for instance, that the application of biofertilizers in combination with chemical fertilizers can have beneficial effects on both plant growth and a reduction in the amount of synthetic fertilizers used [161,163]. 

Only wool and hair are directly applied in the soil without any treatment for keratin recovery. Other animal tissues, such as feathers, hooves, and pig bristles, on the contrary, require a conversion. Small protein hydrolysates recovered from these by-products were demonstrated to have a very good effect on plant physiology and microbiomes [163,164] (in addition, see references listed in Table 4). These hydrolysates can be either produced by bacteria, actinomycetes, and fungi directly in the soil/plant or generated most often through microbial/enzymatic extraction and added to the soil/plant as a liquor/powder [163,164]. Feather hydrolysates, for instance, have been tested in the concentration of 1–3 kg/10 kg soil for cowpea cultivation, 2–5 g/250 g soil for chickpea, 5 g/200 g soil for pea and rice, and 180 mg N/kg soil for lettuce, with very good results [164].

### 5.5. Cosmetics

Cosmetics, together with biomaterials, were one of the first applications of keratin [3,9,165]. As keratin is poorly soluble in water, it cannot be used for cosmetic formulations, such as emulsions, gels, or powders, in its natural form. Apart from the release of specific molecular weight fractions, it should be ensured that keratin-derived products are stable in formulations to avoid unfavorable phenomena such as peptide reaggregation due to hydrophobic interactions [165,166,167]. Keratin hydrolysates have a strong moisturizing action and can preserve the natural hydration of both hair and skin; accordingly, they can increase skin firmness and hair softness [101,167]. The use of keratin hydrolysates can be particularly valuable with increasing age, since, in the body, the keratin content (and other macromolecules of the same family and roles, such as collagen and elastin) gradually decreases. In this case, cosmetic formulations can also help induce the biosynthesis of skin macromolecules [165,167,168]. Wool was one of the first animal by-products to be used for keratin and lipid extraction for incorporation in cosmetic products. Lipids are present in a small amount in wool (1–5%), and they were documented to be a good source of free fatty acids easily absorbed by the skin. However, natural raw materials such as keratin hydrolysate can contain several impurities depending on multiple environmental factors, and safety practices must be ensured by manufacturers to minimize risks to human health [166,168].

### 5.6. Animal Feed

Keratin can be a good source of nutrients for animal feed and for the human diet too. For several decades, feathers, horns, hooves, and hair have been frequently added to animal feed as an inexpensive source of protein [169,170,171]. However, as keratin is insoluble in water and in many digestive fluids, a conversion process is needed to release biologically valuable fractions. Sulfitolysis (NaHSO_3_/LiBr), oxidation (24% *w*/*w* peracetic acid), ionic liquid extraction (with 1-Butyl-3-methylimidazolium chloride), steam explosion (220 °C, 10 min), and domestic microwave extraction in superheated water (180 °C, 150–570 W for up to 7 min) resulted in significantly high yields (40–65%) of keratin hydrolysates for feed use. A recently developed process that combines microwaves with moderately high pressures (15 bar) was able to reach an 85% yield [172]. The amino acid composition of keratin hydrolysates from wool obtained by this process was comparable to other sources, such as soy, whey, bone, and fish isolates; keratin isolates were richer in cysteine, serine, and arginine, while the contents of aspartic and glutamic acids, histidine, and lysine were significantly lower [172]. However, these differences can open the way to more balanced feed by combining several animal feed sources, apart from individual amino acid enrichment [169,170,171]. Feathers are the second most used by-product after wool; their degradation by multiple keratinolytic proteases was demonstrated to be a suitable alternative for alkaline hydrolyses and resulted in higher nutritional quality. Brandelli et al. [132], for instance, reported that *Bacillus licheniformis* can increase feather keratin amino acid digestibility from 30% to 66%. Keratin isolates from animal by-products such as wool have also been tested for their cytotoxicity, with encouraging results [171,172], and these findings can help reduce the strong dependence on other protein sources such as soy and fishmeal.

### 5.7. Energy Devices 

Keratin has recently been studied and applied for the fabrication of renewable fuel cells. These devices are receiving increasing attention as a sustainable technology for electricity generation with negligible CO_2_ emissions [173]. Proton-conductive materials are the core of fuel cells, and natural molecules like proteins and polysaccharides are being explored for both this potential and for their biodegradability [174]. Soon et al. [173] extracted keratin from chicken feathers using 8 M urea and ammonium thioglycolate, at alkaline pH, followed by heating at 60 °C for 6 h. A 10 kDa keratin fraction was obtained, and amyloid fibrils were prepared by heating the keratin isolate at 90 °C in 10% *v*/*v* acetic acid for 5 h. A proton-conductive membrane was fabricated afterward by mixing the fibrils with glyoxal and an acidic compound, followed by thermal curing. The membrane was placed inside a 1cm^2^ cell between two commercial electrodes. The authors noticed that the performance of the keratin membrane is 10–20-fold lower than that of commercial products, but it has the advantage of being more sustainable and less expensive. Hence, further studies can be expected on this potential use of keratin. 

## 6. Environmental and Economic Impact of Keratinous Animal By-Products and of Their Valorization 

Animal by-products rich in keratin represent the unavoidable and inedible livestock residues and are generated in the processing stage. Their environmental impact is associated with the animal processing in the slaughterhouse and their disposal. These two steps represent 5.6–10.7% and 5.7–7.3%, respectively, of the total environmental impact of the livestock (and other) chain (production, processing, retail/distribution, consumption, and disposal), which is estimated to be ca. 2.93 million tonnes of CO_2_-equivalents/million tonnes of food waste [175]. Thus, the emission of ca. 5.12 million tonnes of CO_2_-equivalents can be associated with the 12 million tonnes/year of animal by-products rich in keratin, where the highest share arises from feathers. In this regard, it is worth highlighting that these emissions are forecasted to increase since a global increase in meat protein consumption of 14% is expected in the next decade [39,176]. In addition, in the last twenty years, the composition of the keratinous animal by-product flow has changed. In Europe, livestock husbandry has evolved toward fewer ruminants and pigs, in favor of more poultry species; in this respect, the production of poultry has strongly increased in other parts of the world too. Consequently, the amount of feathers has increased by more than 40% in 2003–2023 [39,176], and the total CO_2_-equivalents in emissions of keratinous by-products have increased accordingly. 

Processes operated for the recovery of keratin and its fractions from animal by-products have different environmental impacts, and this can be a significant driver in choosing one or another, apart from the economic performances and the priorities of application. Biological methods such as microbial or enzymatic hydrolysis have lower environmental and economic impacts than thermal and chemical methods since they are carried out at lower temperatures and for shorter durations. Colantoni et al. [177] estimated that the production of protein hydrolysates by enzymatic hydrolysis requires ca. 66% less fossil energy and emits ca. 60% less CO_2_-equivalents with respect to the thermo-chemical process. However, as a counterpart, the price of specific enzymes, such as keratinases, can be higher than some bulk chemicals such as alkali (NaOH, urea, etc.), acids (HCl, H_2_SO_4_, and others), salts, and buffers. In addition, the composition and weight of protein fractions can be different, thus resulting in differences in performance [178]. The emissions and energy demand of the thermal and thermo-chemical treatments can be reduced by coupling with ultrasonication, and these waves can optimize the bioactivity of protein fractions [172]. Ionic liquids have been demonstrated to be effective in the recovery of keratin and other molecules; however, their status as “green solvents” is debatable. These liquids in fact are thermally very stable and non-volatile, limiting their release into the atmosphere. Yet, their use has increased and has led to the contamination of the terrestrial and aquatic medium, in which these solvents are very toxic. These questions are currently driving several studies on a new generation of biodegradable and safer ionic liquids, some of which derive from amino acids [179].

**Figure 12:**
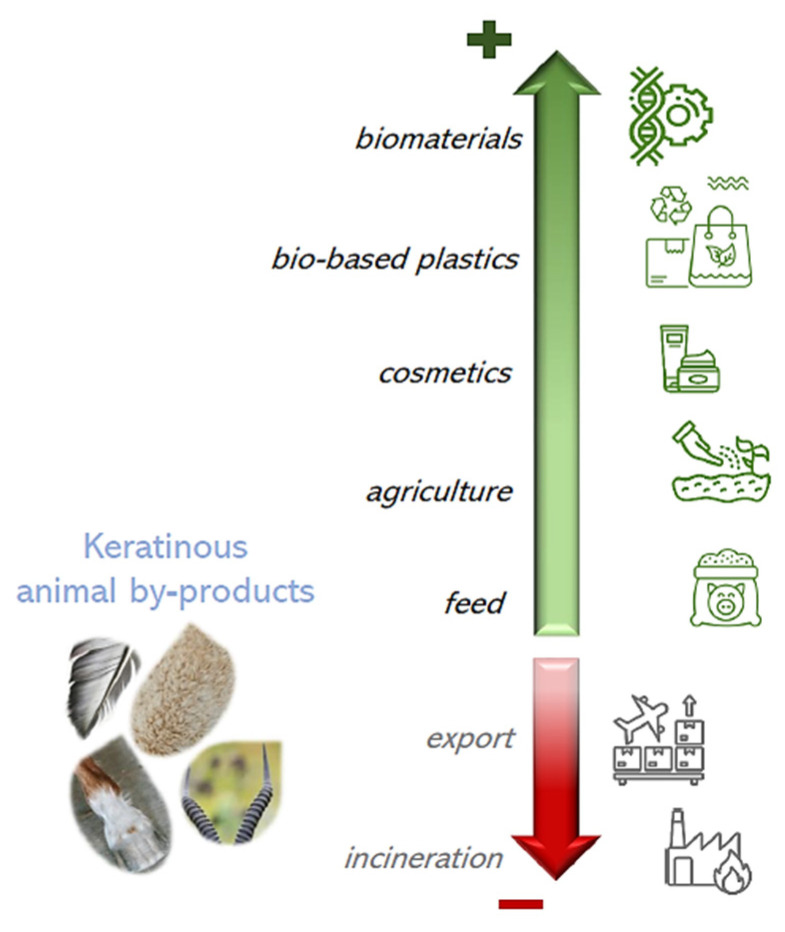
Application of keratin from animal by-products, with increasing economic value (from the red to the green arrow) (image created by authors with pictures from www.pexels.com (accessed on 7 June 2024) and icons made by Pixel perfect from www.flaticon.com (accessed on 7 June 2024)).

The use of keratin for the production of bio-based products, and in particular plastics, could contribute to reducing the environmental impact of conventional petrosourced analogs, in addition to bringing plastics with different functionalities on the market. By a deep analysis of several datasets, Zheng and Suh [180] showed that greenhouse gas (GHG) emissions from the whole life cycle of conventional plastics were 1.5 G tonnes of CO_2_-equivalents in 2015, and this is expected to rise to 6.5 G tonnes by 2050. However, this increase could be avoided by improving the use of biomass in the production of plastics, since bio-plastic emits lower GHG levels than fossil plastics. At present, the production of keratin-based bio-plastics is more expensive than that of plant-based analogs; however, they can have a more positive impact on the environment by avoiding by-product treatment by incineration, and because of their faster absorption in soils [29]. From a socio-economic perspective, the valorization of animal by-products rich in keratin, and other animal by-products, can contribute to the sustainability of the animal production system, increase the income of farmers, limit the dependence on other countries for some critical raw materials like fibers (in the case of the European Union), and create new jobs. Figure 12 summarizes applications of keratin from animal by-products, with an increasing socio-economic impact. 

## 7. Future Trends

Keratin is receiving increasing attention in virtue of its unique biological and mechanical properties and of the availability of important quantities of animal tissues from livestock, which are rich in this natural polymer and are not intended for human consumption. Human hair, although not classified as an animal by-product, is more and more regarded as a renewable biowaste with a great potential for valorization for keratin recovery. The progress made in the last 10–15 years in the knowledge of the in vivo structure of keratin; the assembly of the complex architecture of keratinous animal by-products such as wool, feathers, hooves, horns, pig bristles, and human hair; and extraction processes, in particular biological ones, opens the door to the recovery of keratin at a large scale, for application in bio-based and biomedical products. A significant contribution to a circular bioeconomy and to the reduction in the environmental impact of animal by-products can be foreseen.

## Data Availability

Data are contained within the article.
